# The Host Restriction Factor Interferon-Inducible Transmembrane Protein 3 Inhibits Vaccinia Virus Infection

**DOI:** 10.3389/fimmu.2018.00228

**Published:** 2018-02-16

**Authors:** Chang Li, Shouwen Du, Mingyao Tian, Yuhang Wang, Jieying Bai, Peng Tan, Wei Liu, Ronglan Yin, Maopeng Wang, Ying Jiang, Yi Li, Na Zhu, Yilong Zhu, Tiyuan Li, Shipin Wu, Ningyi Jin, Fuchu He

**Affiliations:** ^1^Key Laboratory of Jilin Province for Zoonosis Prevention and Control, Military Veterinary Institute, Academy of Military Medical Sciences, Changchun, China; ^2^State Key Laboratory of Proteomics, Beijing Proteome Research Center, Institute of Radiation Medicine, Beijing, China; ^3^Jiangsu Co-Innovation Center for Prevention and Control of Important Animal Infectious Diseases and Zoonoses, Yangzhou, China; ^4^2nd Clinical Medical College of Jinan University, Shenzhen People’s Hospital, Shenzhen, China; ^5^State Key Laboratory of Pathogen and Biosecurity, Institute of Microbiology and Epidemiology, Academy of Military Medical Sciences, Beijing, China; ^6^Academy of Animal Science and Veterinary Medicine in Jilin Province, Changchun, China

**Keywords:** interferon-inducible transmembrane protein 3, interferon, vaccinia virus, interferon-stimulated genes, virus entry and binding

## Abstract

Interferons (IFNs) establish dynamic host defense mechanisms by inducing various IFN-stimulated genes that encodes many antiviral innate immune effectors. IFN-inducible transmembrane (IFITM) proteins have been identified as intrinsic antiviral effectors, which block the entry of a broad spectrum of enveloped RNA viruses by interrupting virus-endosomal fusion. However, antiviral activity of IFITM proteins against mammalian DNA virus has not been demonstrated till date. Here, we sought to investigate the antiviral activities and mechanisms of interferon-inducible transmembrane protein 3 (IFITM3) protein against poxvirus infection. Analysis of expression kinetics of cell endogenous IFITM3 protein indicated that vaccinia virus (VACV) infection suppressed its translation, which was independent of IRF3 phosphorylation triggered by VACV. Although silencing of endogenous IFITM proteins did not affect their baseline antiviral effects in the cell, it has reduced the IFN-α-mediated inhibition of VACV infection, and also modulated VACV-induced cell death. Moreover, we discovered that overexpression of IFITM3 significantly restricted VACV infection, replication and proliferation mainly by interfering with virus entry processes prior to the virus nucleocapsid entry into the cytoplasm. Interestingly, IFITM3 overexpression showed an impact on virus binding. Furthermore, IFITM3 interfered with the cytosolic entry of virus through low pH-dependent fashion. Taken together, our findings provide the first evidence of exogenously expressed IFITM3 protein restricting infection of an enveloped DNA virus, thus expanding their antiviral spectrum. This study further explores the complex mechanism and provides novel insights into the interaction between virus infection and host defense.

## Introduction

Interferon-stimulated genes (ISGs) are important integral components of host intrinsic immunity, which can be induced by interferons (IFN) or viral infection and exert antiviral activity at specific stages of the virus replication cycle (1). For instance, Myxovirus resistance (Mx) proteins block the transport of viral RNA to the nucleus through interaction with influenza virus NP and RNA helicases (2). Protein kinase R (PKR) interfere with viral protein synthesis by phosphorylating the α-subunit of eukaryotic translation initiation factor 2α (eIF2α), and tetherin restricts viral release *via* trapping mature viral particles on the plasma membrane (3). Exhilaratingly, IFN-inducible transmembrane (IFITM) proteins were recently reported to restrict the viral entry stage of its lifecycle (4).

Currently, the human *IFITM* family includes five functional genes (*IFITM1, IFITM2, IFITM3, IFITM5*, and *IFITM10*). Among these, *IFITM1, IFITM* 2 and *IFITM3* showed constitutive expression in a wide range of tissues and can be induced by type I and type II IFNs. Meanwhile, it was also noticed that *IFITM5* is not IFN-inducible and is limited to osteoblasts with involvement in bone mineralization, and the function of *IFITM10* remains unclear (5–7). To date, IFITM proteins (mainly referring to IFITM1, 2, and 3) have been reported to restrict the virus entry and infection of several pathogenic enveloped viruses, including influenza A virus (IAV) (8), West Nile virus (WNV) (4), dengue virus (DENV) (9), Ebola virus (EBOV) (10), SARS coronavirus (11), rift valley fever virus (12), Semliki forest virus (13), human immunodeficiency virus type-1 (HIV-1) (14, 15), respiratory syncytial virus (RSV) (16), and Zika virus (17). Interestingly, IFITM3 has been reported to restrict reovirus, a non-enveloped virus (18). In contrast, other studies revealed that IFITM proteins could not inhibit *in vitro* infection with several DNA viruses, such as human cytomegalovirus (HCMV), adenovirus, and human papillomavirus (19). Moreover, no reports have demonstrated the inhibition of DNA viruses, mediated through human IFITM proteins. Notably, IFITM3 protein plays an important role in controlling the infection and pathogenesis of IAV (20, 21), WNV (22), and multiple alphaviruses *in vivo* (23). Although its antiviral mechanism is undefined, IFITM3 has been demonstrated to block virus entry by inhibiting the fusion of viral membrane with the endosomal membrane (24).

Poxviruses, an ancient family of viruses that parasitize invertebrates, birds, reptiles, and mammals, include variola virus (VARV, causative agent of smallpox and a potential weapon of bioterrorism), cowpox virus, vaccinia virus (VACV), monkeypox virus (MPXV, causes smallpox-like fatal disease in humans), and avipoxviruses (25). Among them, VACV is the prototypic poxvirus, a complex and enveloped DNA virus that is closely related to VARV and MPXV (26, 27). Thus, the possibility of zoonotic poxviruses crossing the species barrier to infect other animals or humans may turn out to be a menace to global health status (28–30). VACV serve as a model virus that can provide much information about other poxviruses. Additionally, several attenuated strains, such as modified vaccinia virus Ankara and the VACV Tian Tan (VTT) strain are currently used as vectors for vaccines or gene therapies against various diseases caused by pathogens or cancers, including HIV-1, malaria, and melanoma (31). Due to its biological safety profile and importance in gene therapy, we chose the VTT strain for our study. The membrane of VACV-VTT can fuse with different cellular membranes and is amenable to labeling. Following macropinocytosis or dynamin-mediated fluid phase uptake, VACV particles enter the cells *via* fusion with the plasma membrane at neutral pH or acidified endosomal membrane triggered at low pH (32–34). However, there is no explicit report whether IFITM3 inhibits poxviruses entry by similar mechanisms of exerting its antiviral role in other viruses.

In this study, the role of IFITM3 in vaccinia virus infection was investigated at cellular level and observed that vaccinia virus did not trigger the transcription of IFITM3 or even down regulated IFITM3 expression by an IRF3-independent manner. Surprisingly, silencing of endogenous IFITM3 did not exert any significant effect on virus infection, but it regulated the antiviral activity of type Ι IFN and modulated the vaccinia virus-induced cell death. Interestingly, further evidence suggested that overexpression of IFITM3 could suppress vaccinia virus infection by interfering with virus-cell binding and low pH-dependent virus entry. This study unravels the complex interactions in host-intrinsic defenses against viral infection and further suggests for developing effective antiviral strategies and optimizing the poxvirus-based vaccine vectors.

## Materials and Methods

### Cells and Virus

Human embryonic kidney cells (HEK-293T), baby hamster kidney cells (BHK-21), African green monkey kidney epithelial cells (Vero), human cervical carcinoma cells (HeLa), and lung epithelial (A549) cells were grown in complete DMEM (HyClone) with 10% fetal bovine serum (Thermo) at 37°C in a 5% CO_2_ incubator. A vaccinia virus Tian Tan mutant strain carrying green fluorescent protein gene (VTT-EGFP) was constructed by homologous recombination as described previously (35). VTT strain and VTT-EGFP were propagated in BHK-21 cells, and handed according to the standard biosecurity procedures in a BSL-2 (Biosafety level 2) laboratory and purified on a sucrose gradient as described previously (33). The infectious titer was determined by plaque assay on Vero cells. The concentrations of purified viruses were determined by measuring the optical density (OD) at 260 nm (1 OD = 1.2 × 10^10^ virions) (36).

### Plasmids and Reagents

Human *IFITM3* gene was amplified from the cDNA synthesized from THP-1 cells (ATCC TIB-202). The plasmid pLV-IFITM3 containing human IFITM3 gene with a N-terminal FLAG tag was constructed based on lentivirus-based plasmids, pLV-Puro (Inovogen, Beijing, China). Recombinant Human IFN-α2b was purchased from PeproTech Asia (Rehovot, Israel). Anti-FLAG M2 antibody was purchased from Sigma-Aldrich; anti-IFITM3, anti-GAPDH (glyceraldehyde 3-phosphate dehydrogenase), and anti-β-actin antibodies from Proteintech (Chicago, IL, USA); Anti-VACV D8 (WR113) antibody was purchased from Immune Tech (New York, NY, USA). Anti-GFP and Alexa Fluor 555-conjugated secondary antibodies and 4,6-diamidino-2-phenylindole (DAPI) were purchased from Beyotime (Haimen, China). Long-chain dialkylcarbocyanines (DiD) were procured from Life Technologies (Grand Island, NY, USA).

### Western Blot

HeLa, 293T, or A549 cells were infected with VTT at 0.1 PFU/cell, and after indicated time intervals of infection, the total cell proteins were extracted with RIPA buffer with protease inhibitor cocktail (Roche, Basel, Switzerland). The extracted proteins were electrophoresed by SDS-PAGE, and then electrophoretically transferred to Nitrocellulose blotting membrane (GE Healthcare, Germany). After incubating in blocking buffer (5% bovine serum albumin in Tris-buffered saline) at room temperature for 1 h, the samples of membrane were probed with the indicated specific primary antibodies and corresponding HRP-conjugated IgG secondary antibodies. The bands were then visualized using an enhanced chemiluminescence detection kit (Pierce Biotechnology, Rockford, IL, USA) and X-ray film and quantified using Image J software normalized to GAPDH.

### IFN Sensitivity Assay

HeLa cells grown to 80% confluence in 12-well culture plates were treated with human IFN-α2b (10,000 or 20,000 U/mL) for 24 h and then infected with VTT-EGFP at PFU/cell of 0.1. After 1 h, the medium was removed, and cells were washed and cultured in fresh medium at 37°C. At specified time points, EGFP-positive cells were visualized by fluorescence microscopy and quantified by flow cytometry.

### *IFITM3* Silencing Analysis

The siRNA oligonucleotides targeting IFITM3 and universal negative control siRNA were designed and synthesized from Ribobio (Guangzhou, China), their sequences are: siIFITM3-1: 5′-CCCACGUACUCCAACUUCC [dT][dT]-3′, siIFITM3-2: 5′-UGUCCAAACCUUCUUCUCU [dT][dT]-3′. Cells were transfected with siRNAs (50 nM each well) using RNAiMax (Invitrogen, Carlsbad, CA, USA) following the manufacturer’s instructions. Transfected cells were treated with IFN-α2b (10,000 U/mL) or medium alone for 24 h and then infected with 1 PFU/cell of VTT-EGFP. At 24 h postinfection, the percentage of infected cells was determined by flow cytometry. Alternatively, IFITM3 expression or silencing was confirmed by Western blot.

### Cell Viability Assay

Cells were treated with siRNA for 48 h prior to infection with VTT at PFU/cell of 0.1. At indicated time points, cell viability was assessed by using the MTS/PMS method as described by the manufacturer (Promega, Madison, WI, USA) and determined by measuring the absorbance at 490 nm using a 96-well plate absorbance reader. A time-response curve was created by nonlinear regression analysis. Each assay was designed with six replicates.

### Generation of Stably Expressing Cell Lines

Cells were transfected with pLV-IFITM3 using X-tremeGENE HP DNA transfection reagent (Roche, Basel, Switzerland). After 48 h, cells were selected in complete medium containing 4 µg/mL puromycin (Sigma, Saint Louis, MO, USA). To assess the expression of IFITM3, cell lysates were harvested stably and examined by Western blot analysis with anti-FLAG or IFITM3 antibodies.

### Virus Infection Assays

All cells used in the study were infected with VTT or VTT-EGFP at the indicated multiplicity of infection. The inocula were removed after 1 h and the cells *were* washed with PBS and replaced with fresh complete culture medium. Cells were harvested at various times after inoculation, and virus infection were determined by fluorescence imaging, flow cytometry and quantitative PCR. Alternatively, the infected cell lysates were harvested and examined by Western blot with anti-VACV D8 or EGFP antibodies.

### Plaque Assay

Cells were absorbed with VTT at the designated PFU/cell for 1 h at 4°C, washed, overlaid with complete DMEM medium containing 1% methylcellulose and cultured at 37°C for 2–3 days. Cells were fixed with 10% formaldehyde and plaques were visualized and their counts were estimated with crystal violet staining and subjected to statistical analysis.

### Virus Binding and Entry Assays

Prechilled BHK-IFITM3 or control cells were incubated with 5 MOI of VTT on ice for 1 h to permit virus binding but impede their cell entry. Unbound virus was removed; cells were washed, fixed and processed for immunofluorescence analysis with an anti-D8 antibody and visualized by a confocal laser-scanning microscope (60 × objective lens/oil). Alternatively, virus-bound cell genome was extracted to determine the amount of viral DNA accumulation by quantitative PCR of *EGFP* and VACV *E3L* genes. To assess VACV entry into cells, virus inocula were removed after binding on ice for 1 h, and cells were washed and cultured with the growth medium for 30, 60, 90, and 120 min at 37°C. Total cellular RNA was extracted to measure the relative quantity of VACV early or late gene transcription (*E3L* or *D8*) by qRT-PCR in order to determine the early postentry steps of viral life cycle and infection.

### Virus Membrane Fusion Assay

Vaccinia virus particles were labeled with DiD for 20 min at room temperature in the dark, followed with removal of non-incorporated DiD as described previously (33). DiD-labeled virus particles were incubated with prechilled BHK or 293T cell lines on ice for 1 h. Cells were then washed, trypsinized and then fixed with 4% paraformaldehyde/PBS to quantify using a FACSCalibur flow cytometer.

### Endosomal Acidification Inhibition Assay

Cells were adsorbed with VTT-EGFP at indicated MOIs at 4°C for 1 h. After adsorption, cells were washed with cold PBS to remove unbound virions. Thereafter, the cells were either mock-treated or treated with 10 or 20 mM NH_4_Cl and cultured at 37°C as described previously (32). After 24 h, the infection was analyzed by measuring the EGFP-positive cells with a FACSCalibur flow cytometer.

### RNA Isolation and Quantitative Real-time PCR Analysis

Total cellular RNA was extracted using TRIzol reagent (Life technologies, USA), and then was reverse transcribed with M-MLV reverse transcriptase (Promega) to cDNA. Quantitative real-time PCR was performed using SYBR Green Master Mix (TOYOBO, Japan) on an ABI 7500 Real-Time PCR System (Applied Biosystems, USA). For relative quantitation analysis, samples were normalized based on the expression of the gene encoding human GAPDH as a reference. The specific primers used were as follows:

IFITM3 (forward, 5′-ATGTCGTCTGGTCCCTGTTC-3′ and reverse, 5′-GTCATGAGGATGCCCAGAAT-3′);
VACV D8: (forward, 5′-ATTTTATCTAGACAATTTGCTGCCT-3′and reverse, 5′-CATGATTAGACGACGACAATAGTGT-3′);
VACV E3L: (forward, 5′-TCAGCCATAAGCATCAGCATC-3′ and reverse, 5′-GGATGTCTAAAATCTATATCGACGAACG-3′);
EGFP: (forward, 5′-CATCTTCTTCAAGGACGACG-3′ and reverse, 5′-TGAAGTCGATGCCCTTCAG-3′);
GAPDH: (forward, 5′-ACCCACTCCTCCACCTTTGAC-3′ and reverse, 5′-TGTTGCTGTAGCCAAATTCGTT-3′).

### Membrane Protein Isolation and Evaluation

The membrane proteins from cells were isolated with Minute™ plasma membrane protein isolation kit following the instruction of the manufacturer (Invent Biotechnologies, Eden Prairie, USA). Cells were collected and resuspended with buffer A supplemented with protease inhibitor cocktail (Roche, Basel, Switzerland). The total membrane proteins including organelles and plasma membrane were isolated and evaluated by Western blot as described previously (37), with β-actin as cytosolic marker and CD81 as membrane protein marker.

### Immunofluorescence and Confocal Microscopy

Cells were cultured on glass coverslips, fixed with 4% paraformaldehyde in PBS for 30 min and permeabilized with 0.25% Triton X-100 according to the specific requirement. Blocking was performed for 1 h with 3% BSA in PBS containing 0.3 M glycine. Cells were next incubated with VACV D8 antibody, IFITM3 antibody or LBPA antibody at room temperature for 2 h. After washing, the cells were incubated with anti-mouse IgG conjugated to Alexa Fluor 555 (AF555), anti-rabbit IgG conjugated to FITC and anti-mouse IgG conjugated to Cy3, respectively, at room temperature for 1 h. Intracellular cholesterol was stained with Amplex^®^ Red Cholesterol Assay Kit (Molecular Probes, Inc., Invitrogen) based on the protocol of the manufacturer. Nuclei were stained with DAPI (Invitrogen). Images were detected with a fluorescence microscope or the Leica TCS SP5 Confocal microscope (63 × objective lens).

### Statistical Analysis

Data were calculated as the mean ± SD. Student’s *t*-tests were performed for all analyses using GraphPad Prism 6 software (GraphPad Software Inc., CA, USA). Differences among groups were determined by one-way ANOVA with repeated test. Statistical significance was determined by two-tailed *P* values: *P < 0.05, **P < 0.01, and ***P < 0.001.

## Results

### Vaccinia Virus Infection Downregulates the Expression of IFITM3 Protein

**Figure 1:**
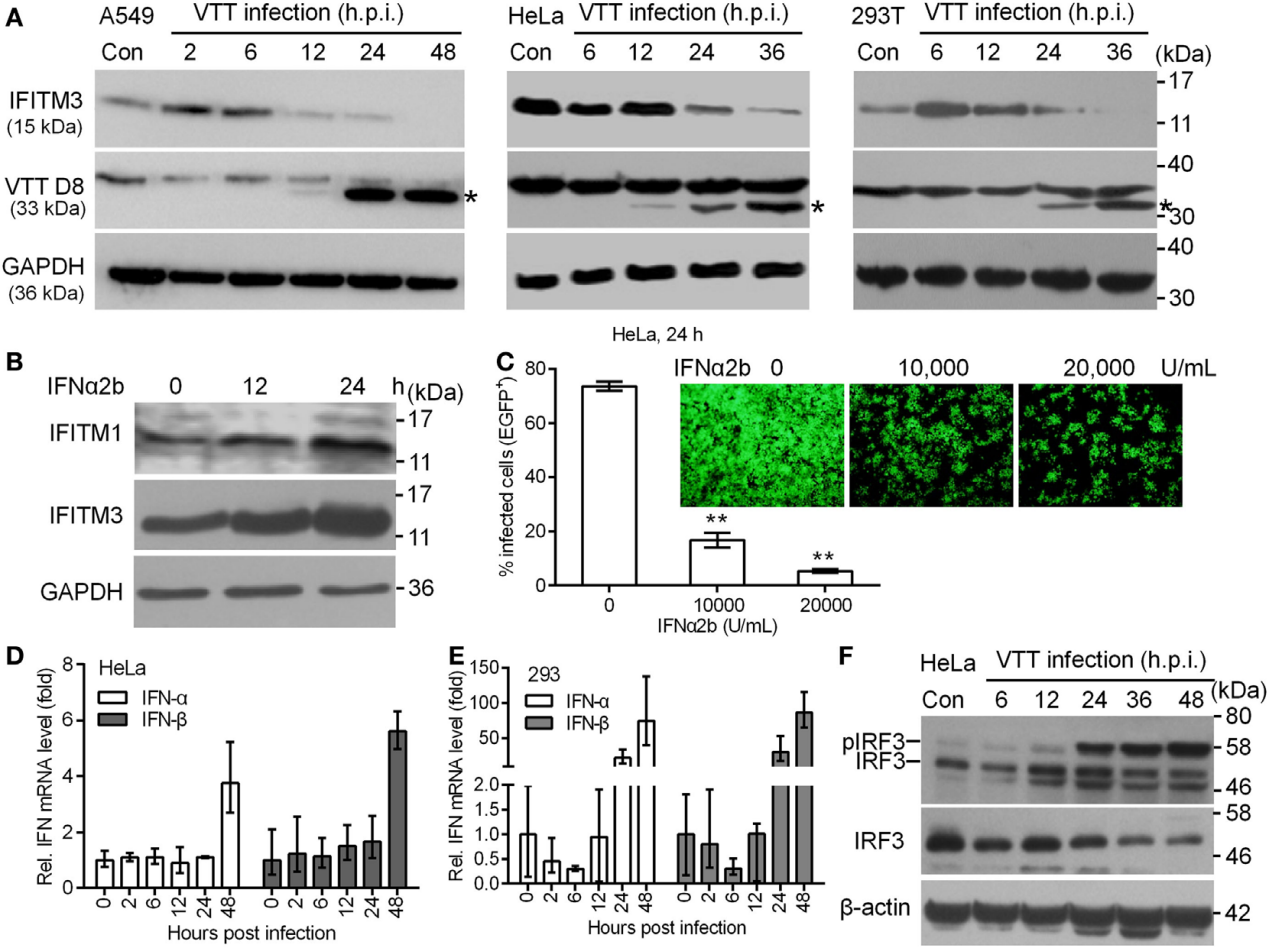
Expression kinetics of endogenous interferon-inducible transmembrane protein 3 (IFITM3) protein modulated by virus infection. **(A)** A549, HeLa, and 293T cells were infected with VACV Tian Tan (VTT) at 0.1 PFU/cell for indicated time points after infection. Total cell proteins were extracted and the expression kinetics of IFITM3 and VTT D8 protein (*) were measured by Western blot and normalized to GAPDH. **(B)** Western blot analysis of IFITM1 and IFITM3 expression in HeLa cells treated with IFNα2b (10,000 U/mL) at indicated time point posttreatment. **(C)** Effect of IFNα2b at indicated concentrations on vaccinia virus infection in HeLa cells evaluated by flow cytometry and fluorescence microscope. **(D,E)** qPCR analysis of IFNα and IFNβ in HeLa and 293T cells infected by VTT infection at indicated time points postinfection. **(F)** Western blot analysis of IRF3 expression and phosphorylation in HeLa cells treated by VTT at indicated time points postinfection. **P<0.01.

To determine the role of endogenous IFITM3 in the interaction between the host cells and vaccinia virus, we first estimated the expression of IFITM3 in HeLa, A549, and 293T cells infected by vaccinia virus, respectively. Notably, we observed that endogenous IFITM3 expression was only transiently and moderately upregulated in A549 and 293T cells but not in HeLa after 2–6 h infection, and then shut down in a timely manner following infection with VACV at 0.1 PFU per cell (Figure 1A) in HeLa, A549, and 293T cells. These findings led us to reinvestigate whether VACV infection affected the signal transduction pathway of ISGs expression. First of all, we found that the expression of IFITM1 or IFITM3 in HeLa cells was further induced following IFNα2b treatment (Figure 1B) and subsequently IFNα2b markedly inhibited VACV infection (Figure 1C). Obviously, this finding indicate that IFNα can induce the activation of signal transduction pathway of ISG expression in HeLa cells and thus exhibit a strong antiviral activity. In order to further investigate how vaccinia virus affected the pathway of type I interferon production in HeLa or 293T cells, the expression of IFNα and IFNβ was measured in mRNA and protein levels. Vaccinia virus could trigger the upregulation of IFNα and IFNβ transcription at 24 or 48 h postinfection (Figures 1D,E), but the corresponding protein expression could not be detected by Western blot. Interestingly, vaccinia virus infection stimulated IRF3 phosphorylation, but simultaneously down regulated its expression in HeLa cells (Figure 1F). However, we were unable to detect STAT1 phosphorylation in HeLa cells infected with vaccinia virus (data not shown). Therefore, we preliminarily judged that HeLa cells are unable to produce type I interferon, and down regulation of IFITM3 is independent of IRF3 phosphorylation activated by vaccinia virus infection. Thus, we speculated that poxviruses had to reprogram host cell translation to benefit the viral life cycle (38). During the viral infection and replication, the majority of host transcripts and its expression into proteins was suppressed or even shut off (39). Therefore, the regulation of expression of IFITM3 as an intrinsic host defense factor that are often induced by virus infection, might also be associated with vaccinia virus.

### Depletion of IFITM3 Modulates the Anti-Poxvirus Effect of IFNα2b and Viral Cytopathogenicity

**Figure 2:**
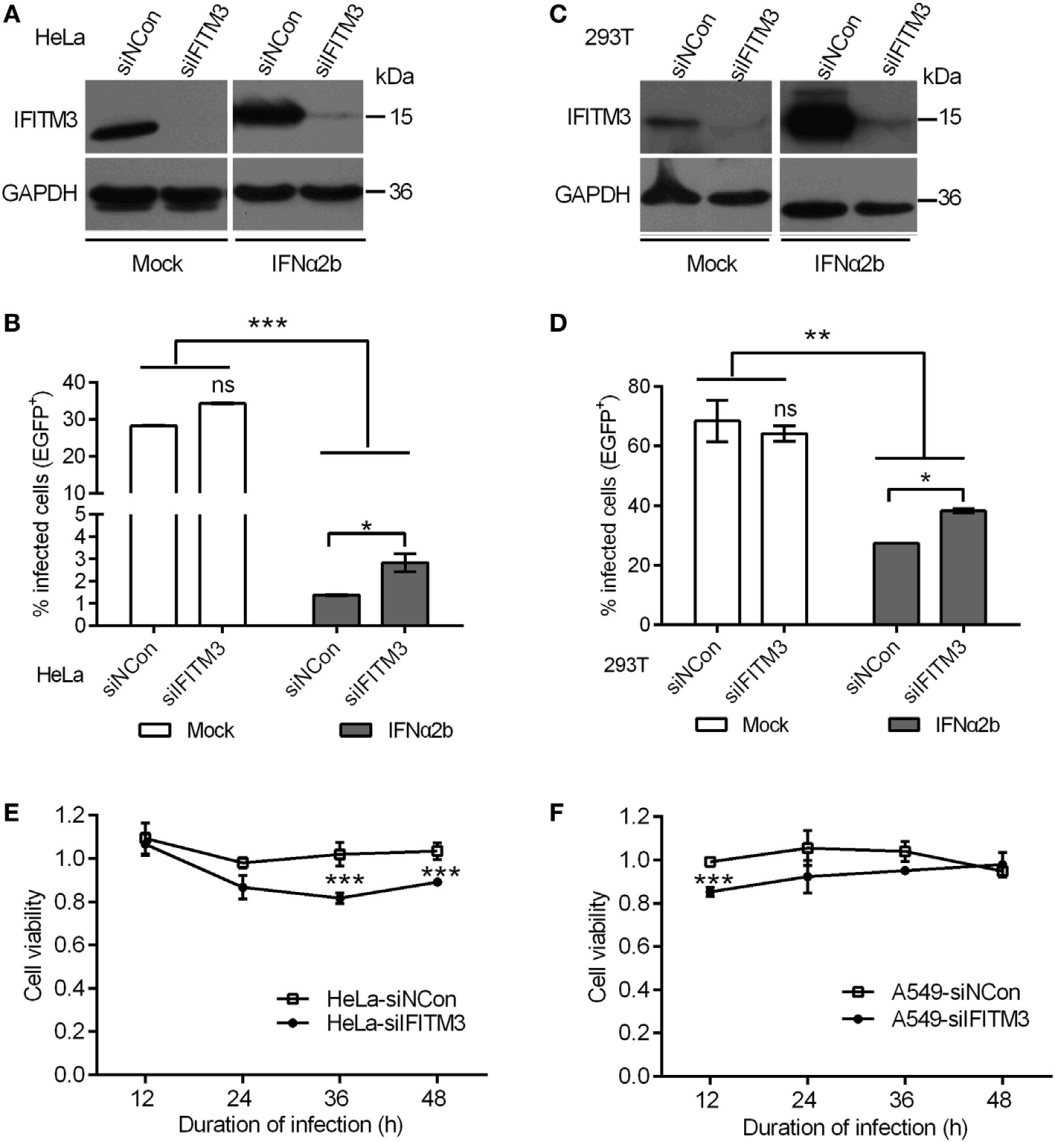
Interferon-inducible transmembrane protein 3 (IFITM3) silencing mediates antiviral actions of IFN-α2b. **(A,C)** IFITM3 expression was analyzed by Western blot in siRNA-transfected HeLa and 293T cells treated with or without IFN-α2b. **(B,D)** Green fluorescent protein (EGFP)-positive cells were scored by flow cytometry of siRNA-transfected HeLa and 293T cells with or without IFN-α2b treatment at 24 h postinfection by vaccinia virus Tian Tan green fluorescent protein (VTT-EGFP). **(E,F)** Viability of siRNA-transfected HeLa and A549 cells infected with VTT virus at 0.1 PFU/cell was assessed by MTS assay. Values represent the mean ± SD, *n* = 4. *P < 0.05, **P < 0.01, and ***P < 0.001.

Earlier studies showed that the depletion of IFITM3 increased IAV (H1N1) infection in primary lung fibroblasts and HeLa cells (4), so we first assessed whether endogenous IFITM3 also interrupts poxvirus infection. In order to accomplish this, the effect of siRNA on the expression of endogenous IFITM3 in HeLa and 293T cells was evaluated, and as expected IFNα2b induced the expression of IFITM3 (Figures 2A,C). In IFNα2b-treated cells, the percentages of infection were significantly suppressed in both HeLa and 293T cells, and knockdown of IFITM3 slightly increased the VTT infection (Figures 2B,D, gray bars). Interestingly, the anti-VACV activity of IFNα2b in HeLa cells was significantly stronger compared to that in 293T cells. However, in the absence of IFNα2b, knockdown of endogenous IFITM3 had no impact on virus infection in both HeLa and 293T cells (Figures 2B,D, black bars). Consistent with other previous studies (40), perhaps VACV had resistance to the inhibition of endogenous IFITM3, which may play a major role in the antiviral effects of interferon.

Although depletion of IFITM3 had no affect on the percentage of infected cells, the effect of IFITM3 on the cytopathogenicity of the virus was uncertain. We therefore investigated whether depletion of IFITM3 had any impact on the viral cytopathogenecity. The siRNA treatments or knockdown of IFITM3 had no significant influence on the cell proliferation (Figures S1A–C in Supplementary Material). Notably, depletion of IFITM3 enhanced the cell death caused by VTT infection (Figures 2E,F), and overexpression of IFITM3 in 293T cells enhanced the cellular antiviral activity (Figures S1D,E in Supplementary Material), indicating that endogenous IFITM3 modulated viral cytopathicity.

### Expression of IFITM3 Restricts VACV Infection in Multiple Cell Lines

**Figure 3:**
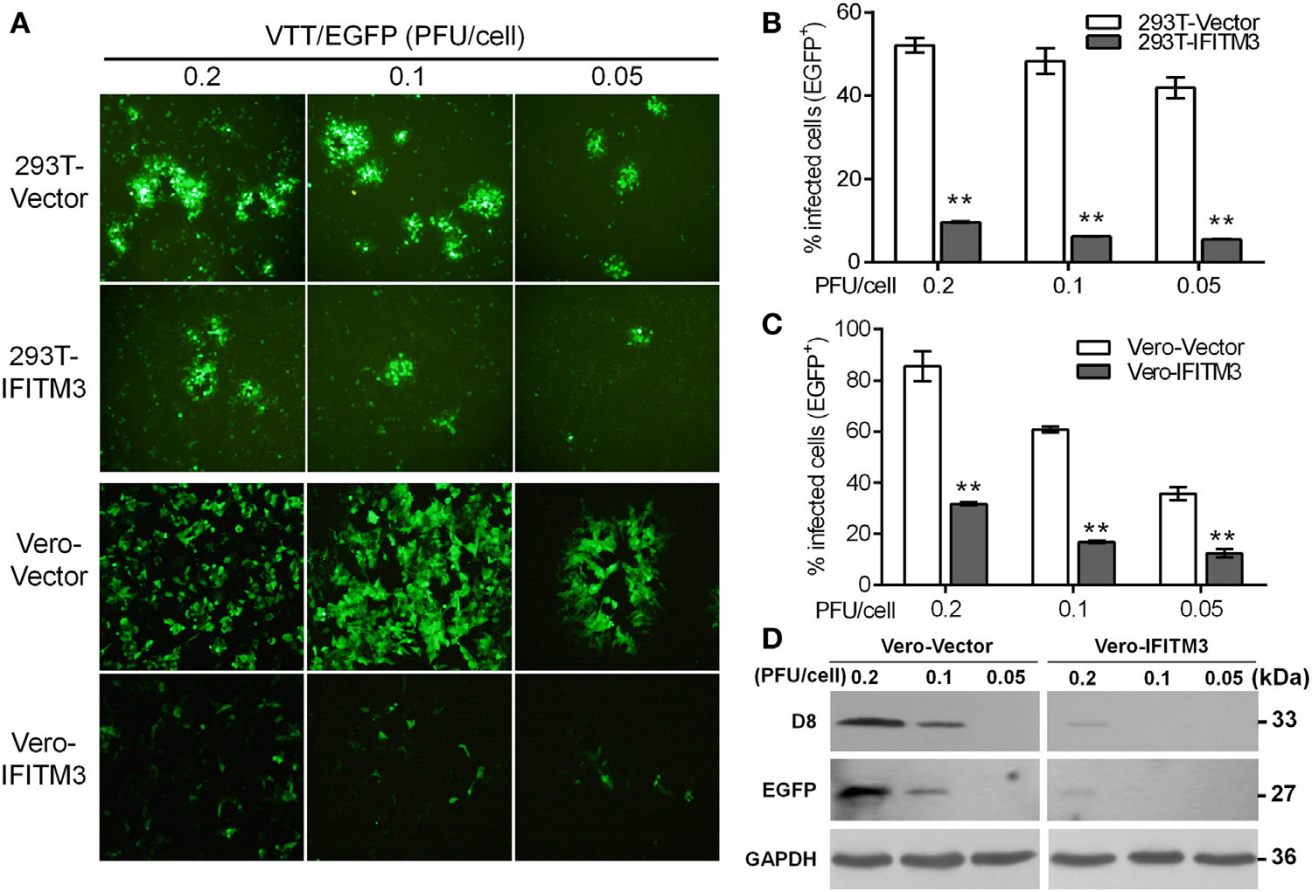
Inhibitory effect of overexpression of interferon-inducible transmembrane protein 3 (IFITM3) on vaccinia virus (VACV) infection. **(A)** Fluorescent plaques formed by vaccinia virus Tian Tan green fluorescent protein (VTT-EGFP) in IFITM3-overexpressed 293T and Vero cells observed *via* fluorescence microscope. **(B,C)** Quantitative analysis of EGFP-positive cells infected by VTT-EGFP was determined by flow cytometry. Values represent the mean ± SD of at least three independent experiments. **P < 0.01. **(D)** Expression of viral D8 and EGFP proteins in Vero cells expressing IFITM3 or vector infected with VTT-EGFP was measured by Western blot.

Our preliminary experiment showed that transient expression of IFITM3 *in vitro* could affect VACV infection (data not shown). In order to precisely evaluate the role of IFITM3 in VACV infection, A549, 293T, HeLa, Vero, and BHK-21 cells stably expressing Flag-N-tagged IFITM3 were generated, and the expression of IFITM3 was analyzed by Western blot (Figure S2A in Supplementary Material). Thereafter, the cells were infected with VTT/EGFP at different multiplicities of infection (PFU/cell) of 0.2, 0.1, and 0.05 for 24 h. We found that the viral cytopathic effect and percentages of infected cells were significantly suppressed by IFITM3 expression in 293T and Vero cells (Figures 3A–C). Furthermore, the infection was also inhibited by the expression of IFITM3 in BHK-21 and A549 cells (Figures S2B,C,E in Supplementary Material). Surprisingly, this restriction role of IFITM3 expression was not observed in HeLa cells (Figures S2D,E in Supplementary Material), and the antiviral activities of IFITM3 expression in 293T, BHK-21, and Vero cells were superior to that in A549 cells against VACV infection. Consistent with the previous report (11), the efficiency of IFITM3-mediated restriction in this study was associated with the cell type and differences in expression. The inhibitory effect of IFITM3 was further confirmed by expression analysis of VTT D8 (cell surface-binding protein) and EGFP reporter in Vero cells simultaneously (Figure 3D). Together, these results showed that IFITM3 expression could inhibit the initial infection and the efficiency of restriction was cell type dependent.

To further address whether IFITM3 could inhibit the VACV replication cycle in host cells, the extent of virus replication in IFITM3-overexpressing cells was analyzed by quantifying the viral gene expression at the transcriptional and translational levels (e.g., viral late gene D8 and inserted gene EGFP) at different time points postinfection with VTT-EGFP. The transcriptional level of viral D8 in IFITM3-positive cells was significantly lower than that in control cells (Figures S3A,E in Supplementary Material). Theoretically, potent inhibition of viral gene transcription should interfere with viral translation. As expected, IFITM3 delayed and blocked the expression of viral D8 protein (Figures S3C,D,F–H in Supplementary Material), suggesting that the exogenous expression of IFITM3 suppressed VACV replication.

**Figure 4:**
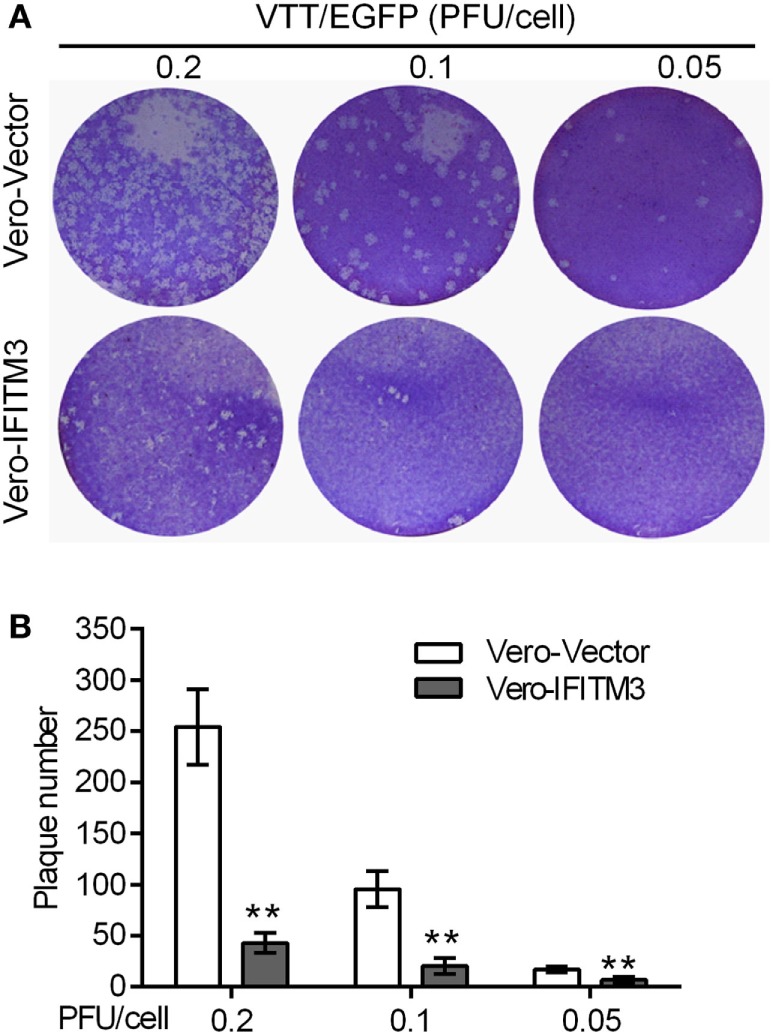
Inhibitory role of interferon-inducible transmembrane protein 3 (IFITM3) on virus cell-to-cell transmission and diffusion. Plaque formation analysis of VACV Tian Tan in Vero-IFITM3 cells at the indicated MOI was performed by crystal violet staining at 48 h postinfection **(A)**, and results are shown as averages of three independent experiments **(B)**. **P < 0.01.

To further confirm the inhibitory role of IFITM3 on virus cell-to-cell transmission and diffusion, plaque formation analysis was carried out by crystal violet staining in Vero cells. The expression of IFITM3 in Vero cells strongly suppressed the cytopathic effect (CPE) and plaque formation of sequential rounds of virus infection (Figure 4).

### IFITM3 Suppresses the Early Stages of VACV Replication

**Figure 5:**
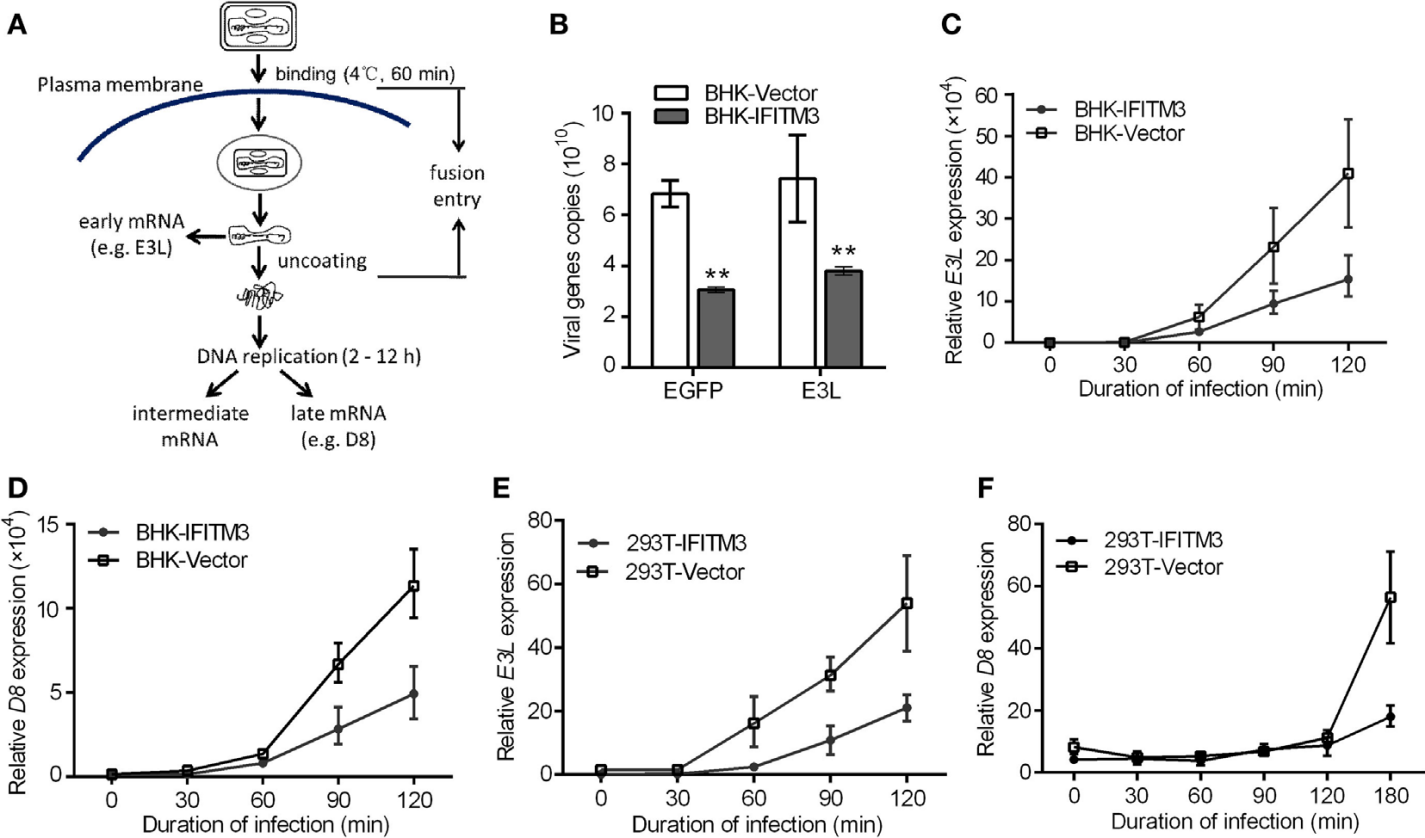
Interferon-inducible transmembrane protein 3 (IFITM3) overexpression restricts VACV Tian Tan (VACV) entry. **(A)** Schematic representation of VACV entry through endocytic pathway. After entry of the VACV core into cells, early genes are expressed, leading to uncoating of the virus core. Subsequently, viral DNA genomes are released, and replication begins within 2–12 h postinfection, which leads to the expression of intermediate and late genes. The transcription of early (*E3L*) or late (*D8*) genes was used to analyze the early stages of viral infection. **(B)** Baby hamster kidney (BHK) cells adsorbed by VACV Tian Tan (VTT) were harvested with a cell scraper, and total cell genomic DNA was extracted for quantification of VTT genomic DNA by qPCR. **(C–F)** IFITM3^+^ BHK and 293T cells were incubated with VTT at 4°C for 1 h, washed with cold PBS and allowed to rest for 0, 30, 60, 90, and 120 or 180 min before total RNA collection. Virus *E3L* **(C,E)** or *D8* **(D,F)** mRNA was quantified by qRT-PCR, and values are presented as the mean ± SD of three independent experiments. *P < 0.05, and **P < 0.01.

Prior studies have revealed that the IFITM3 protein restricts virus infection at the early stage of the viral replication cycle of several RNA virus, such as IAV, WNV, and DENV (4, 11, 24). We therefore speculated that the inhibition of IFITM3 might also occur at the early steps of the VACV lifecycle. To verify this speculation, we first tested the impact of IFITM3 on the initial phase of infection by incubating BHK-IFITM3 with VTT (5 PFU/cell) on ice for 1 h to permit viral binding but with preventing viral entry into the cells (33). Compared with the vector cells, we observed the loss of VTT D8 signal in IFITM3-positive cells at 30, 60, and 90 min postinfection (Figure S4 in Supplementary Material). Interestingly, the D8 signal observed in IFITM3-positive cells seemed to be lower than that in vector cells after adsorption (Figure S4 in Supplementary Material). Next, we exploited the characteristics of the poxvirus replication cycle to evaluate the inhibition of IFITM3 on virus entry. As described in Figure 5A, the VACV early gene transcription and translation occurred within the viral core, and viral DNA replication initiated within a few hours (about 2–12 h) after virus entering the cytoplasm leading to intermediate and late phases of gene expression (34, 36, 38). To verify this speculation, 293T or BHK-21 cells expressing IFITM3 were incubated with VTT (5 PFU/cell) at 4°C for 1 h to permit viral binding (Figure 5A), and the early (e.g., *E3L*) or late (e.g., *D8*) gene transcription was quantified by real-time PCR to serve as an indicator to determine the inhibition efficiency of IFITM3 to VACV entry. Interestingly, we found that expression of IFITM3 inhibited VACV binding (Figure 5B). Moreover, the transcription of the early gene *E3L* and late gene *D8* in IFITM3-expressing cells was delayed and restricted (Figures 5C–F), suggesting that IFITM3-mediated inhibition of VACV infection and replication occurs at the virus entry stage.

### IFITM3 Disturbs VACV Binding

**Figure 6:**
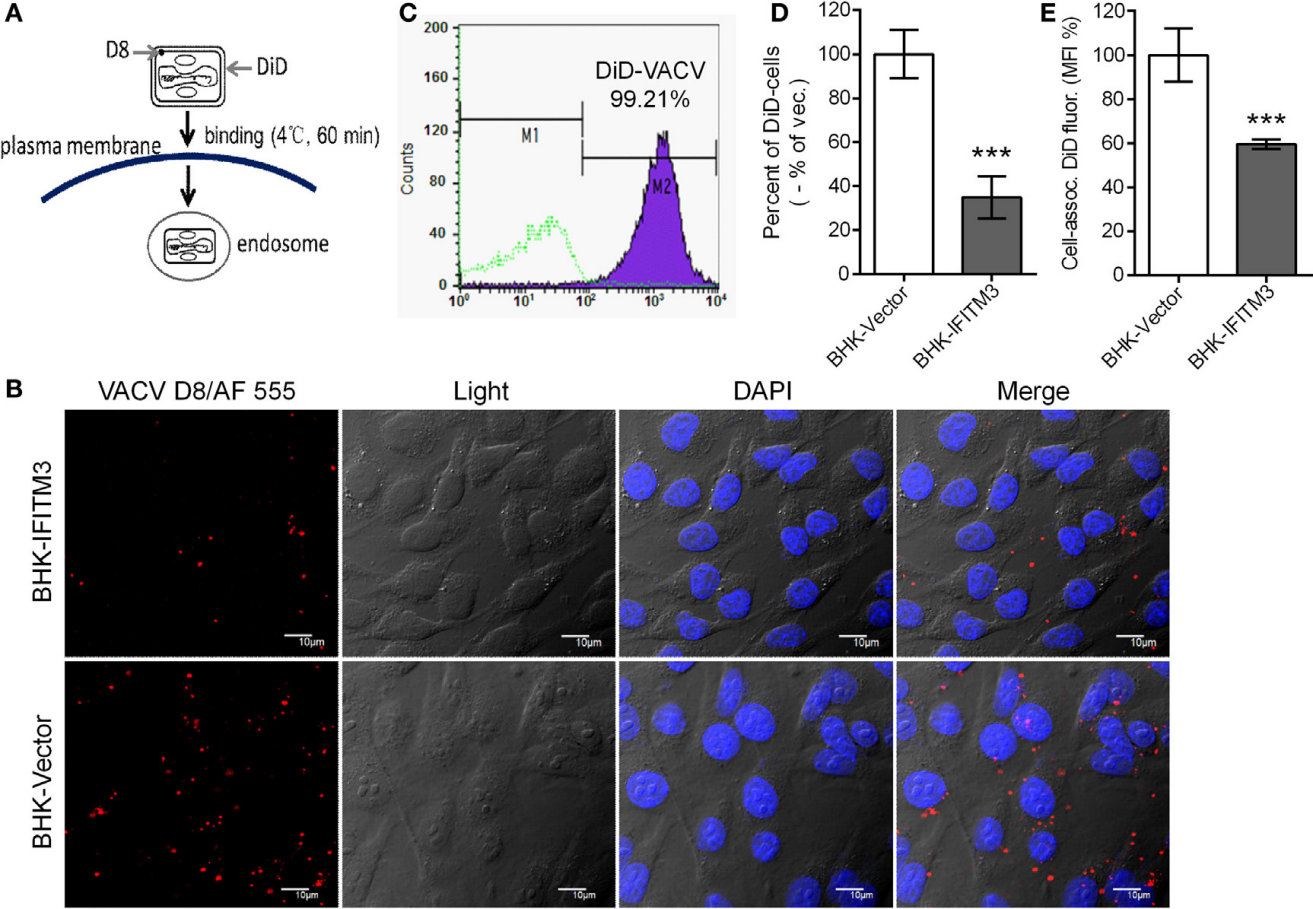
Interferon-inducible transmembrane protein 3 (IFITM3) overexpression disturbs vaccinia virus (VACV)-cell binding. **(A)** Schematic representation of virus attachment analysis by immunostaining of viral D8 protein or using fluorescent probe DiD-labeled virus. **(B)** Baby hamster kidney (BHK) cell lines were incubated with VACV Tian Tan (VTT) at 4°C for 1 h, followed by washing, fixation, immunostaining with anti-D8 antibody (red), staining for DNA (blue) and imaging by confocal laser scanning microscopy. **(C)** VTT particles were labeled with DiD and detected by flow cytometry. **(D,E)** BHK cell lines were incubated with DiD-labeled VTT at MOI of 5 for 1 h on ice, washed, digested and fixed, and DiD-positive cells **(D)** and DiD mean fluorescence intensities **(E)** were quantified by flow cytometry. Values **(D,E)** represent the mean ± SD of three independent experiments. **P < 0.01.

To further determine whether expression of IFITM3 affected VACV attachment, labeling assays with fluorescence-labeled antibody against VACV D8 protein or fluorescent probe DiD to virus particle were performed as mentioned previously in the methods (Figure 6A) (32, 41). Immunofluorescence images suggested that the presence of stably expressed IFITM3 could block viral-cell binding (Figure 6B). To better quantify these effects, purified VTT particles were labeled with DiD, a lipophilic membrane dye, and the labeling rate was determined to be approximately 99.21% (Figure 6C). DiD-labeled virus particles were incubated with IFITM3-positive BHK and vector cells on ice for 1 h, and then DiD-positive cells and DiD mean fluorescence intensities were quantified by flow cytometry. Compared to vector cells, the percentage of IFITM3-positive cells and DiD mean fluorescence (DiD fluor.) intensities (MFI) of IFITM3-positive cells bound with virus particles was significantly lower (Figures 6D,E), indicating that IFITM3 interrupted VTT binding consistent with the data in Figure 5B.

### IFITM3 Co-Localize with LBPA^+^ Late Endosomes and Lysosomes

**Figure 7:**
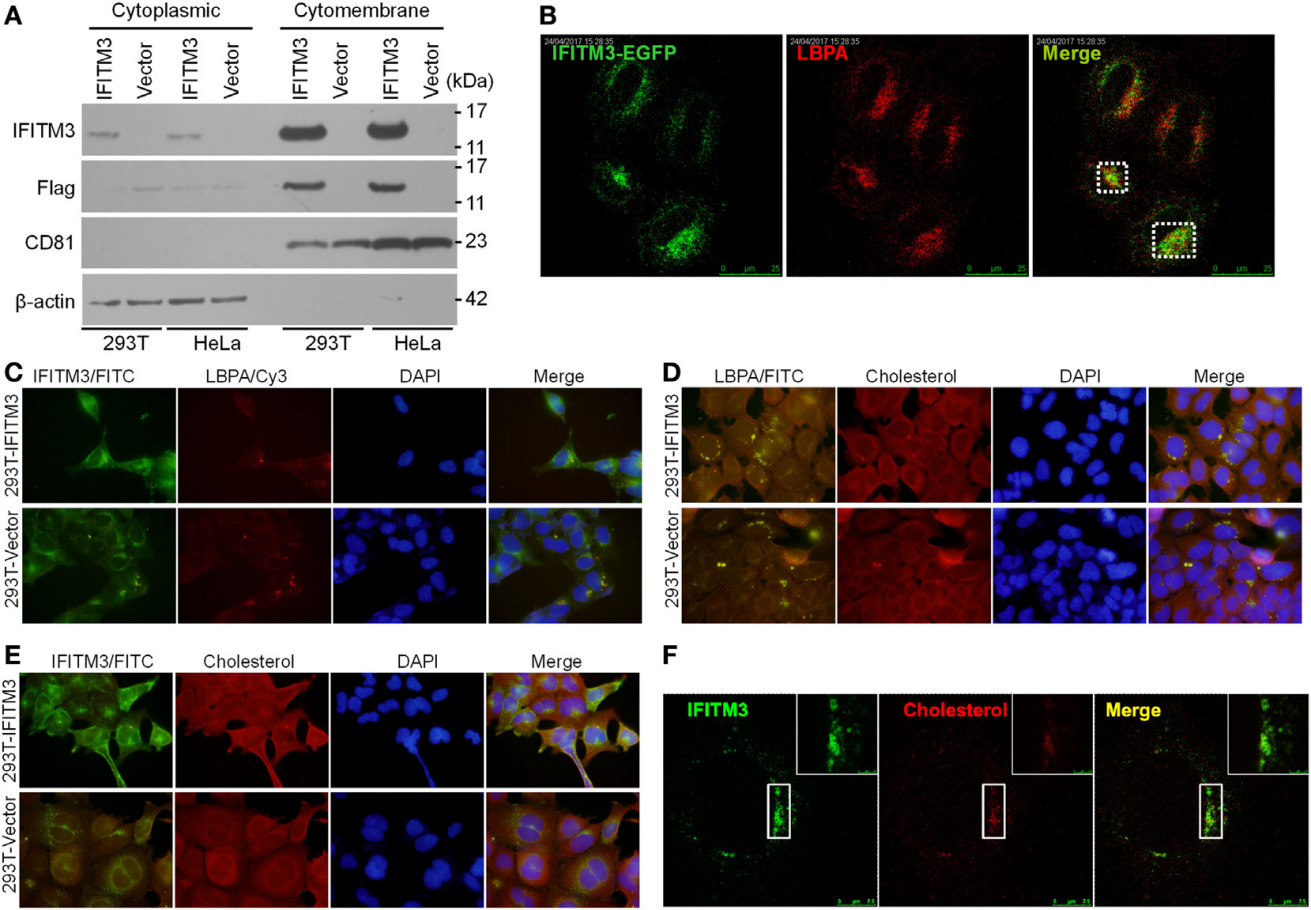
Cellular distribution and localization of interferon-inducible transmembrane protein 3 (IFITM3) protein. **(A)** Cellular distribution of IFITM3 expression in membrane or cytoplasm measured by Western blot analysis following the separation of membrane proteins and plasma proteins of IFITM3^+^ 293T and HeLa cells. CD81 and β-actin were selected as the marker of membrane and cytoplasm proteins, respectively. **(B)** IFITM3 localizes in LBPA^+^ late endosomes evaluated by confocal microscopy. HeLa cells transfected with IFITM3-EGFP expression plasmid for 36 h were stained with mouse monoclonal anti-LBPA followed by a Cy3-conjugated antimouse IgG. **(C–F)** IFITM3 partially localizes in Cholesterol^+^ late endosomes. IFITM3^+^ 293T cells were stained with rabbit anti-IFITM3 and mouse monoclonal anti-LBPA followed by a FITC-conjugated anti-rabbit IgG and Cy3-conjugated anti-mouse IgG, and cholesterol was traced with Amplex^®^ Red Cholesterol Assay Kit. IFITM3 and LBPA, IFITM3 and cholesterol, or LBPA and cholesterol colocalization is evaluated by fluorescence microscope or confocal microscopy. Nucleus is stained with DAPI.

Several reports provided the evidences that IFITM3 is localized in the late endosomal and lysosomal structures (42, 43) with CD63, Rab7, LAMP1, or LAMP2, and thus inhibit the entry of viruses, such as IAV, WNv, and EBOV into these cellular organelles. It was previously reported that the Poxviruses enter the cells either through the plasma membrane at neutral pH or through a low pH-dependent endocytic route (32). In order to elucidate the mechanism of IFITM3 against VACV, we investigated its subcellular localization. IFITM3 was mainly localized in the cell membrane structures by extracting the membrane and cytosol proteins of IFITM3-positive 293T and HeLa cells (Figure 7A). Confocal microscopy revealed that IFITM3 is partially localized in LBPA-positive late endosomes and lysosomes in HeLa cells (Figure 7B). However, LBPA, a phospholipid that has an important role in cholesterol homeostasis, was absent in 293 cells (Figure 7C), whereas, high levels of cholesterol was detected in these cells (Figures 7D,E). Moreover, IFITM3-positive cellular compartments showed much higher degrees of cholesterol (Figure 7F), but the expression of IFITM3 displayed little or no effect on the accumulation of endosomal cholesterol (Figure 7E). Of note, IFITM3 was expressed at cellular membrane lipid raft, and localized at late endosomes and lysosomes, providing a significant correlation of its physical location with its antiviral mechanism.

### Overexpression of IFITM3 Affects pH-Dependent VACV Entry

**Figure 8:**
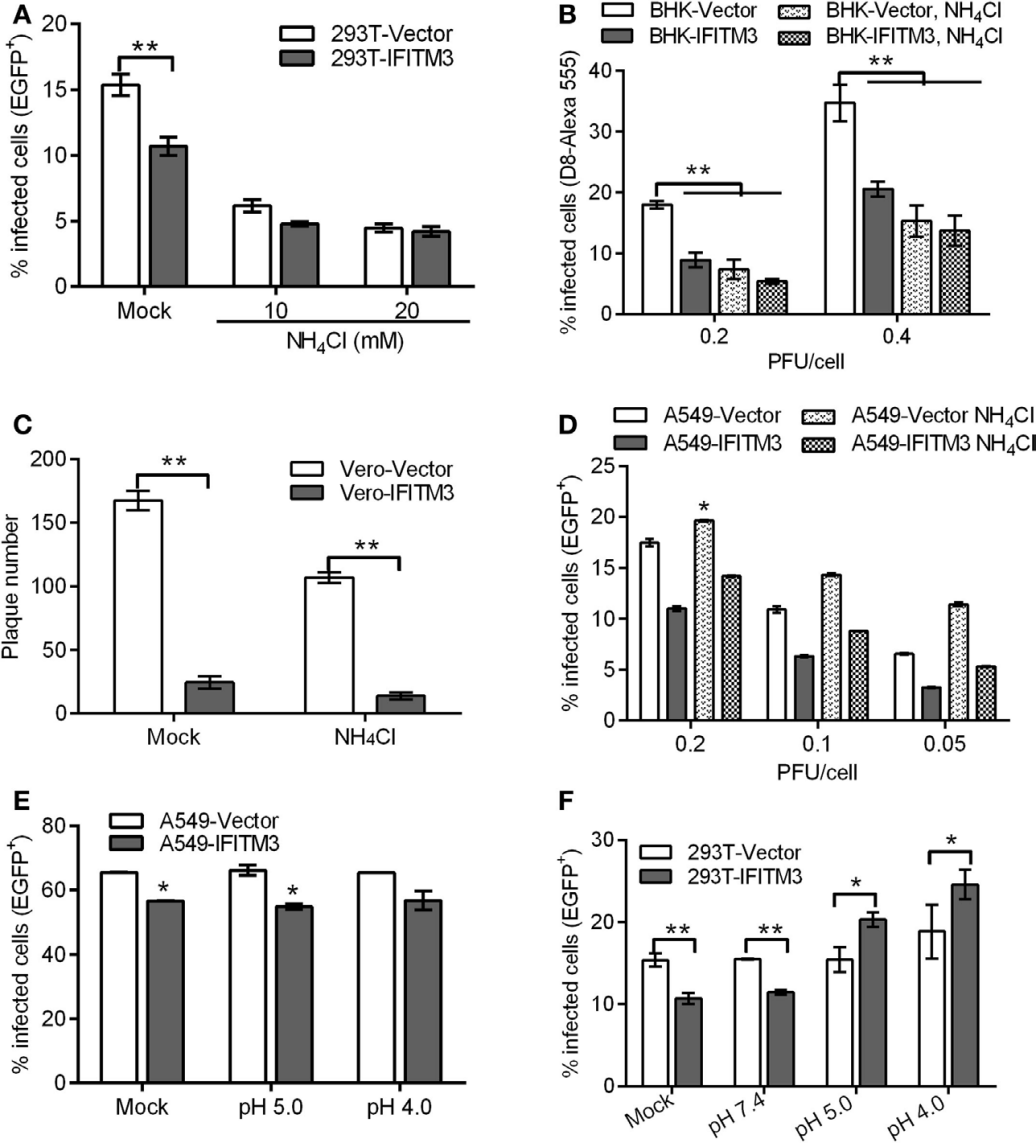
Interferon-inducible transmembrane protein 3 (IFITM3) overexpression affects pH-dependent vaccinia virus (VACV) entry. **(A,B)** IFITM3^+^ 293T and baby hamster kidney cells were infected with vaccinia virus Tian Tan green fluorescent protein (VTT-EGFP) at the indicated MOI on ice for 1 h, followed by either mock treatment or treatment with 10 or 20 mM NH_4_Cl at 37°C for 24 h. Percentages of infected cells were determined by flow cytometry. **(C)** Plaque forming assay of Vero cell lines, which were incubated with VTT at 0.01 MOI on ice for 1 h and then treated with 10 mM NH_4_Cl or buffer (mock) at 37°C for 48 h. **(D–F)** A549 or 293T cells were incubated with VTT-EGFP at the indicated MOI on ice for 1 h and then treated with 10 mM NH_4_Cl or PBS buffer (mock) **(D)** and pH 5.0 buffer, pH 4.0 buffer, or PBS (mock) **(E,F)** at 37°C for 24 h. Percentages of infected cells were determined by flow cytometry. All values represent the mean ± SD, *n* = 3. *P < 0.05 and **P < 0.01.

Since IFITM3 localizes to endosomal compartments including macropinosomes required for VACV entry triggered by a low pH, we further characterized its impact on VACV entry. An endosomal acidification inhibition assay was performed in the absence or presence of ammonium chloride (NH_4_Cl), which neutralizes endosomal pH and consequently prevents the low-pH-dependent activation and triggering of fusion between the viral envelope and endosomal membrane. The results showed that NH_4_Cl treatment reduced the infectivity of VTT by approximately 60% in 293T or BHK cells and inhibited plaque formation of VTT in Vero cells (Figures 8A–C). Moreover, IFITM3-positive cells exhibited lower levels of infection than control cells in the presence of NH_4_Cl, and no difference in the percentages of IFITM3-positive cells infected by VTT-EGFP was observed in the presence or absence of NH_4_Cl (Figures 8A,B). These results suggested that IFITM3 did not affect VACV entry in a low-pH independent pathway. Therefore, we assumed that IFITM3 might exert antiviral activity during viral endosomal trafficking. However, the infection of A549 cells with VTT did not show any significant affect by NH_4_Cl (Figure 8D), perhaps suggesting that VACV infects A549 cells mainly in a pH-independent fusion. To understand this observation, A549 cell lines were incubated with virions at 4°C for 1 h to permit attachment but with preventing viral entry. The cells were then exposed briefly to buffer at pH 7.4, pH 5.0, or pH 4.0 for 5 min before incubation with regular medium at neutral pH for 24 h. The results showed that the infection of VTT in A549 cells was not accelerated by low pH, and had no affect the antiviral activity of IFITM3 (Figure 8E), indicating that VTT may enter A549 cells either through the plasma membrane or a neutral pH endocytic route. However, we performed a similar experiment in 293T cell-derived lines and found an enhancement of infection in 293T cells following low-pH treatment of cell-binding virions in the presence or absence of IFITM3. Moreover, the percentage of infected 293T cells overexpressing IFITM3 was higher than that of vector control cells infected with VTT-EGFP (Figure 8F). These results indicated that a low pH could antagonize the antiviral activity of IFITM3 in 293T cells. Taken together, these findings suggested that IFITM3 could mainly block the cytosolic entry of the low pH-dependent virus.

## Discussion

The IFITM proteins, an important IFN-induced family of effector molecules, have been identified as important host restriction factors for the control of infection by several pathogenic enveloped and non-enveloped viruses. In particular, IFITM3 was demonstrated to play a central role in limiting IAV entry and replication *in vitro* and in *IFITM3* knockout mice (4, 20, 21). Moreover, previous studies also revealed that the IFITM3 protein targets viral fusion that relies on low pH in late endosomes or lysosomes (8, 24, 44). While we have a good understanding of the antiviral activities of IFITMs against RNA viruses especially the enveloped viruses that enter the host cells *via* pH-dependent endocytosis, much less is known about the activity of IFITMs to DNA viruses. First, we observed that the expression of endogenous IFITM3 was downregulated after infection with VACV but upregulated by IAV strains, such as H5N1 or H9N2 (data not shown). Perhaps VACV infection harnesses the host cell protein expression system competitively, and helps to switch on virus protein synthesis after shut down of host mRNA or protein synthesis. Consistent with this concept, VACV infection has been shown to interfere with RNA synthesis and processing (45, 46) and impaired host protein translation (47). However, little is known currently about what or how vaccinia virus infection makes an impact on host gene expression. Alternatively, based on the pathogen–host evolutionary relationship, perhaps VACV infection triggers the machineries required for evading host defense effectively and then suppresses or degrades levels of IFN-induced factors or other host antiviral proteins for viral replication. Recently, for instance, it is reported that TRIM29 as a negative regulator of innate immune activation inhibited type I interferon production by inducing K48-linked ubiquitination of STING for protein degradation, leading to double-strand DNA virus escape from host immune defense (48). In our research, we found vaccinia virus downregulated the expression of interferon stimulating genes. However, how TRIM29 function in the innate immune suppression induced by vaccinia virus is unclear. Whether TRIM29 is related to the expression downregulation of interferon stimulating genes including IFITMs by vaccinia virus need to be explored. Even though several DNA viruses, such as HCMV (49) and herpes simplex virus (HSV) (50) have demonstrated the ability to induce expression of IFITM3 (1), these proteins could not inhibit HCMV entry and infection (19). These observations indicated that a complex interactive relationship between virus and host cells is largely dependent on the cell type and virus strain.

Our data provided a clear support that, the expression of IFITM3 protein in different epithelioid cells restricts the infection and replication of the VACV strain VTT, an old smallpox vaccine strain, as judged by the percentages of infected cells, diameter and quantity of plaque formation, transcription and translation of viral protein. However, none of these proteins were found to have an effect on VACV replication in a loss-of-function screen by genome-scale RNA interference (40). Coincidentally, we performed similar experiments in HeLa, A549, or 293T cells, which constitutively expressed IFITM3 protein. The depletion of endogenous IFITM3 protein in HeLa and 293T cells had no significant effect on infection by VACV (Figures 2B,D). This result is most likely due to the compensatory mechanism of host cells against virus infection. Interestingly, the silencing of endogenous IFITM3 protein enhanced the cell death caused by VTT infection (Figures 2E,F), suggesting that IFITM3 may have a role in mediating resistance to virus infection.

Antiviral factors, including IFITM, IFIT, and MxA which mediate intrinsic antiviral immunity, are preexisting in general to serve as first-line defenders by restricting initial virus infection and are upregulated by IFNs or viral infections (1). The silencing of IFITM proteins *in vitro* has resulted in increased infection by multiple enveloped or non-enveloped viruses, and knockout of *IFITM* genes *in vivo* could accelerate pathogenesis (20, 21, 51). The depletion of IFITM proteins also led to loss of IFN antiviral activity by about 40–70% to IAV (4), indicating that these effectors are critical for IFN-mediated innate immunity. Consistent with this notion, we observed that the silencing of IFITM3 protein resulted in diminution of IFNα triggered protection from VACV infection in HeLa and 293T cells (Figure 2). These findings contributed to our understanding of cell- or virus-dependent functions of IFITM proteins in natural infection and innate immunity.

Although the actual mechanisms remain unclear, previous studies have demonstrated that IFITM3 protein restricted infection by blocking cytosolic entry of viruses utilizing the endocytosis pathway (4, 5), and also by affecting viral-endosomal membrane fusion to block viral nucleocapsid release (24, 44). Consistent with this notion, we observed that IFITM3 had restricted and delayed the transcription of early or late genes at early steps of the VACV life cycles, suggested that blocking of IFITM3 occurs prior to the transcriptional initiation of early genes. The early transcription of VACV is restricted and confined to the virion core, and entry of the core into the cytoplasm results in the mRNA production of early genes, which ceases when the core is disassembled (52, 53). Thus, IFITM3 inhibits VACV infection by preventing cytosolic entry of the virus core. Previous studies showed that entry of VACV MV particles was cell-type-dependent and occurred *via* direct fusion with the plasma membrane or a pH-dependent endosomal pathway (54, 55). Here, we analyzed the role of IFITM3 in low pH-triggered viral entry, and found that it is most likely restricted through the low pH-dependent route but not the low pH-independent route of VACV entry. Additionally, we also tested the effect of low pH treatment on IFITM3-mediated restriction. The findings showed that low pH treatment promoted virus infection but displayed an antagonistic effect on the restricted role of IFITM3. However, the detailed mechanism remains to be elucidated further.

Surprisingly, our finding showed that human IFITM3 affected VACV-cell binding, which was inconsistent with previous studies, besides a recent report that showed an inhibition of sIFITM3 at the attachment phase of FDMV (56). We observed the localization of IFITM3 on the cellular surface following overexpression, which had complemented the previous observation that IFN treatment or overexpression modified IFITM localization (57). Coincidentally, Compton et al. also observed an increase in IFITM2 and IFITM3 proteins on the cell surface following IFN-α2a treatment (15). Although the molecular mechanism of restriction needs further study, our report provides insights into the IFITM3-mediated restriction of VACV entry and indicates a potentially new mechanism to explain the antiviral activity of IFITMs.

In conclusion, this study provides further understanding of the antiviral spectrum of IFN-induced IFITM proteins and the complex interactions between virus infection and host cells. Although IFITM proteins have been demonstrated to protect cells from diverse enveloped RNA virus infections by inhibiting virus-cell fusion, our study gives evidence for their antiviral activity against a DNA virus by interfering with VACV-cell binding and restricting virus core entry. Simultaneously, we also analyzed the role of endogenous IFITM proteins in IFN-mediated innate immunity against virus infections. These results encourage researchers to explore the potential application of IFITM proteins as viral entry inhibitors and to further analyze VACV entry pathways and the mechanism of IFITM3-mediated restriction.

## Author Contributions

CL, SD, NJ, and FH conceived and designed the experiments. CL and SD performed the experiments. MT, YW, JB, PT, WL, RY, MW, YJ, YL, NZ, YZ, Tl, and SW contributed reagents/materials/analysis tools. CL, SD, NJ, and FH analyzed the data and wrote the article.

## Conflict of Interest Statement

The authors declare that the research was conducted in the absence of any commercial or financial relationships that could be construed as a potential conflict of interest.

## References

[1] Yan N Chen ZJ. Intrinsic antiviral immunity. Nat Immunol (2012) 13(3):214–22. 10.1038/ni.2229 22344284 PMC3549670

[2] Yu WC Chan RW Wang J Travanty EA Nicholls JM Peiris JS Viral replication and innate host responses in primary human alveolar epithelial cells and alveolar macrophages infected with influenza H5N1 and H1N1 viruses. J Virol (2011) 85(14):6844–55. 10.1128/JVI.02200-10 21543489 PMC3126566

[3] Neil SJ. The antiviral activities of tetherin. Curr Top Microbiol Immunol (2013) 371:67–104. 10.1007/978-3-642-37765-5_3 23686232

[4] Brass AL Huang IC Benita Y John SP Krishnan MN Feeley EM The IFITM proteins mediate cellular resistance to influenza A H1N1 virus, West Nile virus, and dengue virus. Cell (2009) 139(7):1243–54. 10.1016/j.cell.2009.12.017 20064371 PMC2824905

[5] Smith S Weston S Kellam P Marsh M. IFITM proteins-cellular inhibitors of viral entry. Curr Opin Virol (2014) 4:71–7. 10.1016/j.coviro.2013.11.004 24480526 PMC7185728

[6] Lewin AR Reid LE McMahon M Stark GR Kerr IM. Molecular analysis of a human interferon-inducible gene family. Eur J Biochem (1991) 199(2):417–23. 10.1111/j.1432-1033.1991.tb16139.x 1906403

[7] Moffatt P Gaumond MH Salois P Sellin K Bessette MC Godin E Bril: a novel bone-specific modulator of mineralization. J Bone Miner Res (2008) 23(9):1497–508. 10.1359/jbmr.080412 18442316

[8] Desai TM Marin M Chin CR Savidis G Brass AL Melikyan GB. IFITM3 restricts influenza A virus entry by blocking the formation of fusion pores following virus-endosome hemifusion. PLoS Pathog (2014) 10(4):e1004048. 10.1371/journal.ppat.1004048 24699674 PMC3974867

[9] Chan YK Huang IC Farzan M. IFITM proteins restrict antibody-dependent enhancement of dengue virus infection. PLoS One (2012) 7(3):e34508. 10.1371/journal.pone.0034508 22479637 PMC3316688

[10] Wrensch F Karsten CB Gnirss K Hoffmann M Lu K Takada A Interferon-induced transmembrane protein-mediated inhibition of host cell entry of ebolaviruses. J Infect Dis (2015) 212(Suppl 2):S210–8. 10.1093/infdis/jiv255 26034199 PMC4564551

[11] Huang IC Bailey CC Weyer JL Radoshitzky SR Becker MM Chiang JJ Distinct patterns of IFITM-mediated restriction of filoviruses, SARS coronavirus, and influenza A virus. PLoS Pathog (2011) 7(1):e1001258. 10.1371/journal.ppat.1001258 21253575 PMC3017121

[12] Mudhasani R Tran JP Retterer C Radoshitzky SR Kota KP Altamura LA IFITM-2 and IFITM-3 but not IFITM-1 restrict Rift Valley fever virus. J Virol (2013) 87(15):8451–64. 10.1128/JVI.03382-12 23720721 PMC3719792

[13] Weston S Czieso S White IJ Smith SE Wash RS Diaz-Soria C Alphavirus restriction by IFITM proteins. Traffic (2016) 17(9):997–1013. 10.1111/tra.12416 27219333 PMC5025721

[14] Lu J Pan Q Rong L He W Liu SL Liang C. The IFITM proteins inhibit HIV-1 infection. J Virol (2011) 85(5):2126–37. 10.1128/JVI.01531-10 21177806 PMC3067758

[15] Compton AA Bruel T Porrot F Mallet A Sachse M Euvrard M IFITM proteins incorporated into HIV-1 virions impair viral fusion and spread. Cell Host Microbe (2014) 16(6):736–47. 10.1016/j.chom.2014.11.001 25464829 PMC7104936

[16] Zhang W Zhang L Zan Y Du N Yang Y Tien P. Human respiratory syncytial virus infection is inhibited by IFN-induced transmembrane proteins. J Gen Virol (2015) 96(Pt 1):170–82. 10.1099/vir.0.066555-0 25228491

[17] Savidis G Perreira JM Portmann JM Meraner P Guo Z Green S The IFITMs inhibit Zika virus replication. Cell Rep (2016) 15(11):2323–30. 10.1016/j.celrep.2016.05.074 27268505

[18] Anafu AA Bowen CH Chin CR Brass AL Holm GH. Interferon-inducible transmembrane protein 3 (IFITM3) restricts reovirus cell entry. J Biol Chem (2013) 288(24):17261–71. 10.1074/jbc.M112.438515 23649619 PMC3682530

[19] Warren CJ Griffin LM Little AS Huang IC Farzan M Pyeon D. The antiviral restriction factors IFITM1, 2 and 3 do not inhibit infection of human papillomavirus, cytomegalovirus and adenovirus. PLoS One (2014) 9(5):e96579. 10.1371/journal.pone.0096579 24827144 PMC4020762

[20] Bailey CC Huang IC Kam C Farzan M. Ifitm3 limits the severity of acute influenza in mice. PLoS Pathog (2012) 8(9):e1002909. 10.1371/journal.ppat.1002909 22969429 PMC3435252

[21] Everitt AR Clare S Pertel T John SP Wash RS Smith SE IFITM3 restricts the morbidity and mortality associated with influenza. Nature (2012) 484(7395):519–23. 10.1038/nature10921 22446628 PMC3648786

[22] Gorman MJ Poddar S Farzan M Diamond MS. The interferon-stimulated gene Ifitm3 restricts West Nile virus infection and pathogenesis. J Virol (2016) 90(18):8212–25. 10.1128/JVI.00581-16 27384652 PMC5008082

[23] Poddar S Hyde JL Gorman MJ Farzan M Diamond MS. The interferon-stimulated gene IFITM3 restricts infection and pathogenesis of arthritogenic and encephalitic alphaviruses. J Virol (2016) 90(19):8780–94. 10.1128/JVI.00655-16 27440901 PMC5021394

[24] Feeley EM Sims JS John SP Chin CR Pertel T Chen LM IFITM3 inhibits influenza A virus infection by preventing cytosolic entry. PLoS Pathog (2011) 7(10):e1002337. 10.1371/journal.ppat.1002337 22046135 PMC3203188

[25] Diven DG. An overview of poxviruses. J Am Acad Dermatol (2001) 44(1):1–16. 10.1067/mjd.2001.109302 11148468

[26] Shchelkunov SN. An increasing danger of zoonotic orthopoxvirus infections. PLoS Pathog (2013) 9(12):e1003756. 10.1371/journal.ppat.1003756 24339772 PMC3855571

[27] Di Giulio DB Eckburg PB. Human monkeypox: an emerging zoonosis. Lancet Infect Dis (2004) 4(1):15–25. 10.1016/S1473-3099(04)01140-5 14720564 PMC9628772

[28] Orba Y Sasaki M Yamaguchi H Ishii A Thomas Y Hang’ombe BM Orthopoxvirus infection among wildlife in Zambia. J Gen Virol (2014) 96:390–4. 10.1099/vir.0.070219-0 25319753

[29] Bera BC Shanmugasundaram K Barua S Venkatesan G Virmani N Riyesh T Zoonotic cases of camelpox infection in India. Vet Microbiol (2011) 152(1–2):29–38. 10.1016/j.vetmic.2011.04.010 21571451

[30] Osadebe LU Manthiram K McCollum AM Li Y Emerson GL Gallardo-Romero NF Novel poxvirus infection in 2 patients from the United States. Clin Infect Dis (2014) 60(2):195–202. 10.1093/cid/ciu790 25301210 PMC5854477

[31] Verardi PH Titong A Hagen CJ. A vaccinia virus renaissance: new vaccine and immunotherapeutic uses after smallpox eradication. Hum Vaccin Immunother (2012) 8(7):961–70. 10.4161/hv.21080 22777090 PMC3495727

[32] Townsley AC Weisberg AS Wagenaar TR Moss B. Vaccinia virus entry into cells via a low-pH-dependent endosomal pathway. J Virol (2006) 80(18):8899–908. 10.1128/JVI.01053-06 16940502 PMC1563910

[33] Laliberte JP Weisberg AS Moss B. The membrane fusion step of vaccinia virus entry is cooperatively mediated by multiple viral proteins and host cell components. PLoS Pathog (2011) 7(12):e1002446. 10.1371/journal.ppat.1002446 22194690 PMC3240603

[34] Mercer J Helenius A. Vaccinia virus uses macropinocytosis and apoptotic mimicry to enter host cells. Science (2008) 320(5875):531–5. 10.1126/science.1155164 18436786

[35] Du S Li C Wang Y Liu C Ren D Li Y Construction and evaluation of a new triple-gene expression cassette vaccinia virus shuttle vector. J Virol Methods (2012) 185(2):175–83. 10.1016/j.jviromet.2012.06.022 22766182

[36] Earl PL Moss B Wyatt LS Carroll MW Generation of recombinant vaccinia viruses. Current Protocols in Molecular Biology. New York: John Wiley & Sons, Inc (1998). p. 16.7.1–9 10.1002/0471142727.mb1617s43 18265124

[37] Zhu X He Z Yuan J Wen W Huang X Hu Y IFITM3-containing exosome as a novel mediator for anti-viral response in dengue virus infection. Cell Microbiol (2015) 17(1):105–18. 10.1111/cmi.12339 25131332 PMC7162390

[38] Liem J Liu J. Stress beyond translation: poxviruses and more. Viruses (2016) 8(6):169. 10.3390/v8060169 27314378 PMC4926189

[39] Schnierle BS Moss B. Vaccinia virus-mediated inhibition of host protein synthesis involves neither degradation nor underphosphorylation of components of the cap-binding eukaryotic translation initiation factor complex eIF-4F. Virology (1992) 188(2):931–3. 10.1016/0042-6822(92)90556-5 1585660

[40] Beard PM Griffiths SJ Gonzalez O Haga IR Pechenick Jowers T Reynolds DK A loss of function analysis of host factors influencing vaccinia virus replication by RNA interference. PLoS One (2014) 9(6):e98431. 10.1371/journal.pone.0098431 24901222 PMC4047015

[41] Carter GC Law M Hollinshead M Smith GL. Entry of the vaccinia virus intracellular mature virion and its interactions with glycosaminoglycans. J Gen Virol (2005) 86(Pt 5):1279–90. 10.1099/vir.0.80831-0 15831938

[42] Narayana SK Helbig KJ McCartney EM Eyre NS Bull RA Eltahla A The interferon-induced transmembrane proteins, IFITM1, IFITM2, and IFITM3 inhibit hepatitis C virus entry. J Biol Chem (2015) 290(43):25946–59. 10.1074/jbc.M115.657346 26354436 PMC4646249

[43] Bailey CC Zhong G Huang IC Farzan M. IFITM-family proteins: the cell’s first line of antiviral defense. Annu Rev Virol (2014) 1:261–83. 10.1146/annurev-virology-031413-085537 25599080 PMC4295558

[44] Shen C Wu XR Jiao WW Sun L Feng WX Xiao J A functional promoter polymorphism of IFITM3 is associated with susceptibility to pediatric tuberculosis in Han Chinese population. PLoS One (2013) 8(7):e67816. 10.1371/journal.pone.0067816 23874452 PMC3706438

[45] Pedley S Cooper RJ. The inhibition of HeLa cell RNA synthesis following infection with vaccinia virus. J Gen Virol (1984) 65(Pt 10):1687–97. 10.1099/0022-1317-65-10-1687 6208316

[46] Becker Y Joklik WK Messenger RNA in cells infected with vaccinia virus. Proc Natl Acad Sci U S A (1964) 51:577–85. 10.1073/pnas.51.4.577 14166765 PMC300120

[47] Rice AP Roberts BE. Vaccinia virus induces cellular mRNA degradation. J Virol (1983) 47(3):529–39. 6620463 10.1128/jvi.47.3.529-539.1983 PMC255294

[48] Xing J Zhang A Zhang H Wang J Li XC Zeng MS TRIM29 promotes DNA virus infections by inhibiting innate immune response. Nat Commun (2017) 8(1):945. 10.1038/s41467-017-00101-w 29038422 PMC5643338

[49] Zhu H Cong JP Shenk T. Use of differential display analysis to assess the effect of human cytomegalovirus infection on the accumulation of cellular RNAs: induction of interferon-responsive RNAs. Proc Natl Acad Sci U S A (1997) 94(25):13985–90. 10.1073/pnas.94.25.13985 9391139 PMC28419

[50] Nicholl MJ Robinson LH Preston CM. Activation of cellular interferon-responsive genes after infection of human cells with herpes simplex virus type 1. J Gen Virol (2000) 81(Pt 9):2215–8. 10.1099/0022-1317-81-9-2215 10950979

[51] Wang Z Zhang A Wan Y Liu X Qiu C Xi X Early hypercytokinemia is associated with interferon-induced transmembrane protein-3 dysfunction and predictive of fatal H7N9 infection. Proc Natl Acad Sci U S A (2014) 111(2):769–74. 10.1073/pnas.1321748111 24367104 PMC3896201

[52] Broyles SS. Vaccinia virus transcription. J Gen Virol (2003) 84(Pt 9):2293–303. 10.1099/vir.0.18942-0 12917449

[53] Yang Z Bruno DP Martens CA Porcella SF Moss B. Simultaneous high-resolution analysis of vaccinia virus and host cell transcriptomes by deep RNA sequencing. Proc Natl Acad Sci U S A (2010) 107(25):11513–8. 10.1073/pnas.1006594107 20534518 PMC2895082

[54] Whitbeck JC Foo CH Ponce de Leon M Eisenberg RJ Cohen GH. Vaccinia virus exhibits cell-type-dependent entry characteristics. Virology (2009) 385(2):383–91. 10.1016/j.virol.2008.12.029 19162290 PMC4041486

[55] Schmidt FI Bleck CK Mercer J. Poxvirus host cell entry. Curr Opin Virol (2012) 2(1):20–7. 10.1016/j.coviro.2011.11.007 22440962

[56] Xu J Qian P Wu Q Liu S Fan W Zhang K Swine interferon-induced transmembrane protein, sIFITM3, inhibits foot-and-mouth disease virus infection in vitro and in vivo. Antiviral Res (2014) 109:22–9. 10.1016/j.antiviral.2014.06.008 24973762 PMC7113896

[57] Perreira JM Chin CR Feeley EM Brass AL. IFITMs restrict the replication of multiple pathogenic viruses. J Mol Biol (2013) 425(24):4937–55. 10.1016/j.jmb.2013.09.024 24076421 PMC4121887

