# Supplementary material for: The Host Restriction Factor Interferon-Inducible Transmembrane Protein 3 Inhibits Vaccinia Virus Infection
Source: Front Immunol. 2018 Feb 16;9:228. doi: 10.3389/fimmu.2018.00228 (PMC5820317; doi:10.3389/fimmu.2018.00228)
Supplement: Supplementary file 1 [file Presentation_1.PDF]

Supplemental Material

The host restriction factor IFITM3 Inhibits Vaccinia Virus Infection

Chang Li[1,2,3,6#]* , Shouwen Du[1,6 #], Mingyao Tian[1#], Yuhang Wang[1], Jieying Bai[1] , Peng Tan[1], Wei Liu[4], Ronglan Yin[5], Maopeng Wang[1], Ying Jiang[2], Yi Li[1], Na Zhu[1], Yilong Zhu[1], Tiyuan Li[6], Shiping Wu[6], Ningyi Jin[1,3,6]*, Fuchu He [2]*

[#] These authors contributed equally to this article and are co-first authors.

* Correspondence: Corresponding Author: ningyij@126.com ＆ hefc@nic.bmi.ac.cn

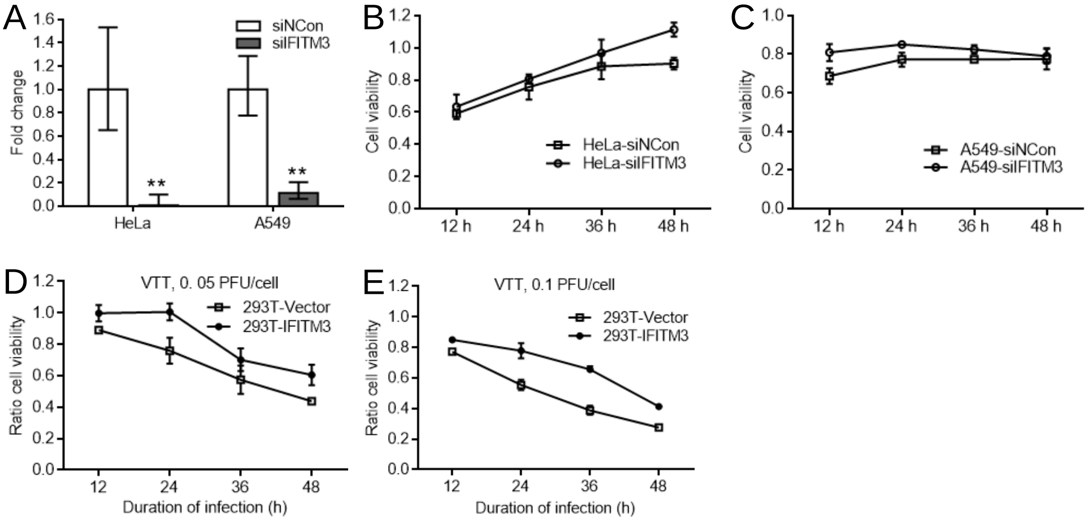

FIGURE S1 | IFITM3 silencing had no influence on cell proliferation and viability and overexpression of IFITM3 in 293T cells enhanced the cellular antiviral activity. (A) HeLa and A549 cells were transfected with IFITM3 siRNA or control siRNA, and then silencing of IFITM3 was assessed by RT-qPCR. **(B, C)** Viability of siRNA-transfected HeLa and A549 cells was evaluated by MTS assay. (**D, E**) Viability of IFITM3-overexpressing 293T cells infected with VTT virus at 0.1 PFU/cell was assessed by MTS assay. In all cases, values represent the mean ±SD of at least three independent experiments.

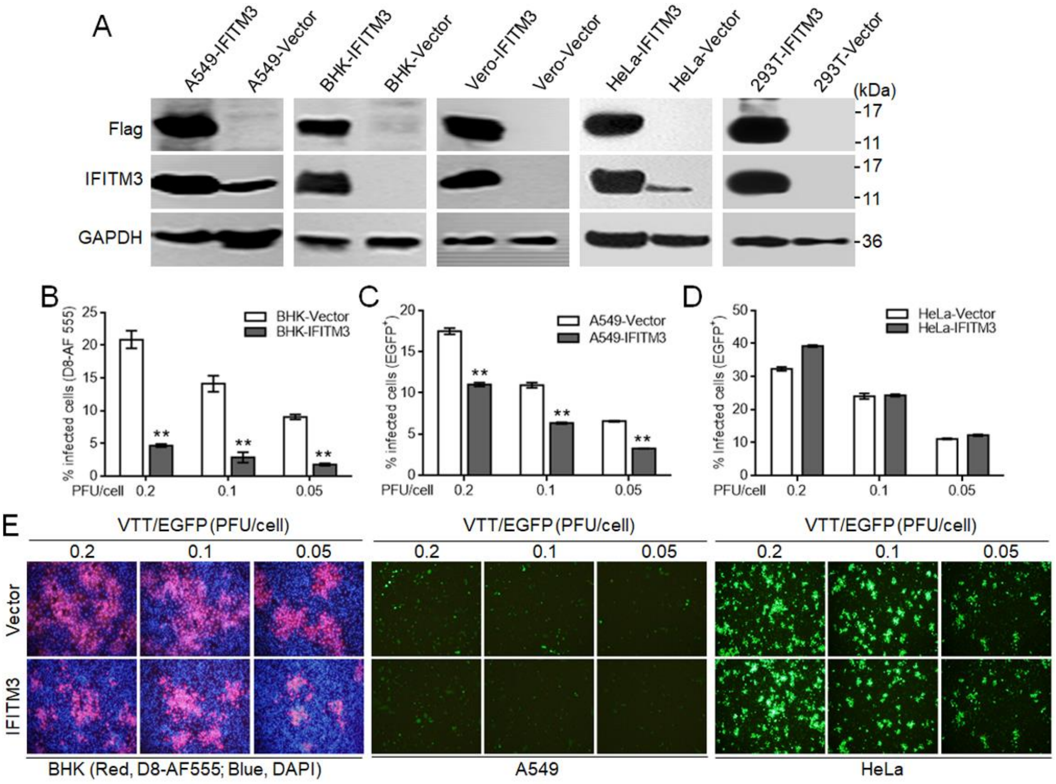

FIGURE S2 | IFITM3 overexpression inhibits VACV infection in multiple cell lines. (A) IFITM3 protein expression levels in A549, BHK-21, Vero, HeLa and 293T cells were measured by Western blot with anti-Flag and anti-IFITM3 antibodies. GAPDH was included as a loading control. **(B-D)** Quantitative analysis of EGFP-positive cells infected by VTT-EGFP was determined by flow cytometry. BHK, A549 and HeLa cells stably expressing IFITM3 or vector alone were infected with VTT-EGFP at the indicated PFU/cell for 24 h or 36 h, infected cells were evaluated for the expression of EGFP by flow cytometry. Values represent the mean ± SD of at least three independent experiments. $^{**}P < 0.01$. (**E**) Representative images of IFITM3-positive or Vector control cells infected with VTT the indicated PFU/cell and stained to detect the viral D8 (red) and cell nuclei (blue) or observed the EGFP-expressing cells via fluorescence microscope (100 ×).

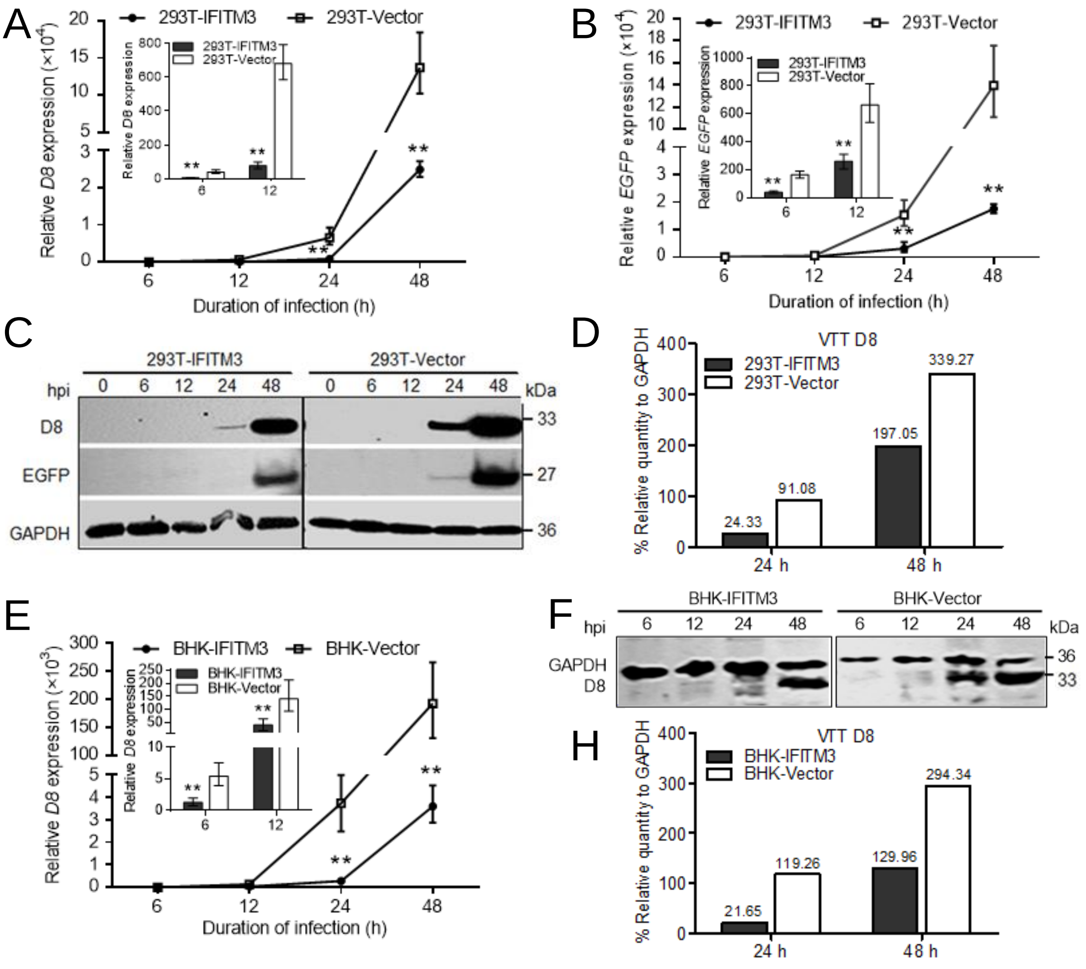

FIGURE S3 | IFITM3 suppresses VACV replication and delays virus transcription and translation. Cells expressing IFITM3 or vector alone were incubated with VTT-EGFP for 1 h at 4 ℃ and then maintained in growth medium. At indicated time points post-infection, cells were harvested to determine intracellular viral D8 (**A** and **E**) or EGFP (**B**) mRNA and D8 protein (**C**, **D**, **F** and **H**) levels by RT-qPCR and Western blot, respectively, normalized to GAPDH. All values represent the mean ± SD of three independent experiments. *$P < 0.05$, **$P < 0.01$.

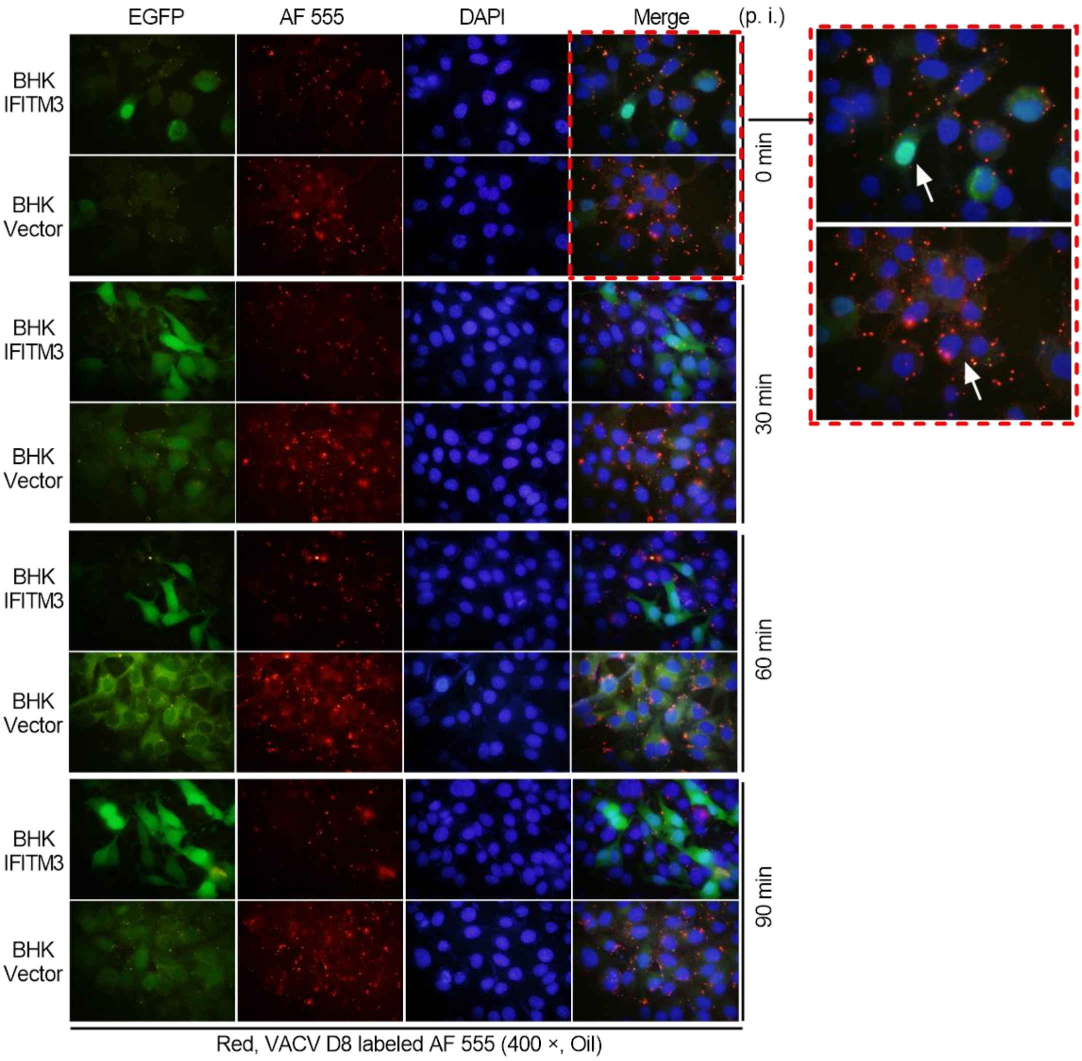

FIGURE S4 | IFITM3 over-expression restricts VACV entry. BHK-IFITM3 or vector cells were incubated with 5 PFU/cell of VTT on ice for 1 h to permit virus attachment, washed and cultured for 0, 30, 60 and 90 min. Cells were then fixed, immunostained with an anti-D8 antibody (red) and Alexa Fluor 555 (AF555) –conjugated anti-Mouse IgG, stained for DNA (blue) and imaged by fluorescence microscopy (400 ×, oil). Images are representative of two independent experiments.
